# P-396. Can ‘Handshake Stewardship’ Decrease Multi-Drug Resistant Healthcare-Associated Infections and All-Cause Mortality? - Insights from A South Indian Tertiary Hospital

**DOI:** 10.1093/ofid/ofae631.597

**Published:** 2025-01-29

**Authors:** Aleena Issac, V Sreeraj, Deljo Puthoor, V K Prathibha, V Geethalakshmi, Maridas Tom Thomas, Thomas Joseph, Gopika P Mohan, Mariya Johnson, Alga P Thomas, Sneha Santhosh, P R Lubaina

**Affiliations:** Amala Institute of medical sciences Thrissur , Pathanamthitta, Kerala, India; Amala Institute of Medical Sciences, Thrissur, Kerala, India; Amala Institute of Medical Sciences, Thrissur, Kerala, India; Amala Institute of Medical Sciences, Thrissur, Kerala, India; Amala Institute of Medical Sciences, Thrissur, Kerala, India; Amala Institute of Medical Sciences, Thrissur, Kerala, India; Amala Institute of Medical Sciences, Thrissur, Kerala, India; Amala Institute of Medical Sciences, Thrissur, Kerala, India; Amala Institute of Medical Sciences, Thrissur, Kerala, India; Amala Institute of Medical Sciences, Thrissur, Kerala, India; Amala Institute of Medical Sciences, Thrissur, Kerala, India; Amala Institute of Medical Sciences, Thrissur, Kerala, India

## Abstract

**Background:**

Multi drug resistant Healthcare Associated Infections (MDR HAI) pose significant challenges for both patients and healthcare facilities, often stemming from inappropriate antibiotic usage. 'Handshake Stewardship' involves a proactive approach of auditing and providing feedback on antibiotic prescriptions to ensure their rationality, with discussions held confidentially with treating physicians. Endorsed by the Centers for Disease Control and Prevention and The Joint Commission, this method aims to engage clinicians in addressing clinical barriers to antimicrobial stewardship.

**Figure:**
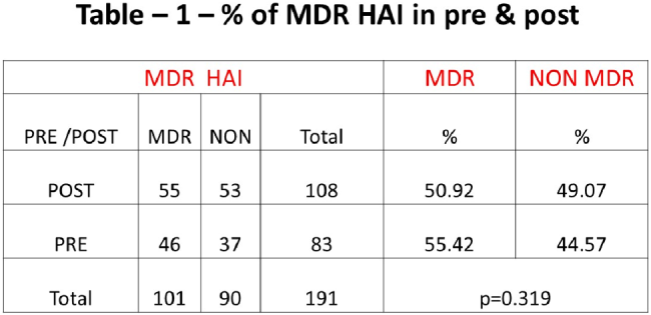

**Methods:**

This study compares MDR HAI in the inpatients of two timelines, pre period May 2021 to April 2022 and post period May 2022 to April 2023. In the post period, the rationality of the antibiotic therapy is discussed with the clinician (in-person approach) by the Antimicrobial stewardship pharmacists within 24 hours of starting the monitored antibiotic. The clinician may or may not accept the interventions and the outcome was documented electronically. The primary outcome was the MDR HAI. Antimicrobial susceptibility testing was performed by the Clinical and Laboratory Standards Institute and ‘MDR’ was assigned as per the joint initiative document by the European Centre for Disease Prevention and Control (ECDC) and the Centers for Disease Control and Prevention (CDC). HAI referred from the CDC’s National Healthcare Safety Network (NHSN) criteria. The secondary outcome was all-cause mortality. Descriptive cross-tabulations and chi-square tests (for all Categorical variables) were performed.

**Figure:**
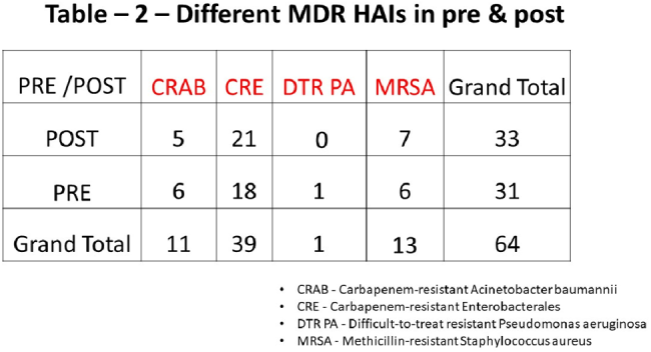

**Results:**

83 HAIs in the pre phase period has 46 cases with MDR status in the culture report, whereas the post era reported 55 MDR status among 108 HAI cases. The post era has less percentage compared to the pre era with no statistical significance (p=0.319) (table -1). The MDR types depicted in table-2. It was found that all-cause mortality in MDR HAI showed no statistically significant difference (Pearson Chi-Square = 0.246) between pre and post phase.

**Conclusion:**

'Handshake Stewardship' did not lead to a significant reduction in MDR HAIs or all-cause mortality during the study period, further long-term evaluations are warranted to assess its effectiveness comprehensively.

**Disclosures:**

**All Authors**: No reported disclosures

